# Bitcoin and S&P500: Co-movements of high-order moments in the time-frequency domain

**DOI:** 10.1371/journal.pone.0277924

**Published:** 2022-11-22

**Authors:** Elie Bouri, Ladislav Kristoufek, Nehme Azoury

**Affiliations:** 1 School of Business, Lebanese American University, Beirut, Lebanon; 2 The Czech Academy of Sciences, Institute of Information Theory and Automation, Prague, Czech Republic; 3 Business School, Holy Spirit University of Kaslik, Jounieh, Lebanon; Beihang University, CHINA

## Abstract

Interactions between stock and cryptocurrency markets have experienced shifts and changes in their dynamics. In this paper, we study the connection between S&P500 and Bitcoin in higher-order moments, specifically up to the fourth conditional moment, utilizing the time-scale perspective of the wavelet coherence analysis. Using data from 19 August 2011 to 14 January 2022, the results show that the co-movement between Bitcoin and S&P500 is moment-dependent and varies across time and frequency. There is very weak or even non-existent connection between the two markets before 2018. Starting 2018, but mostly 2019 onwards, the interconnections emerge. The co-movements between the volatility of Bitcoin and S&P500 intensified around the COVID-19 outbreak, especially at mid-term scales. For skewness and kurtosis, the co-movement is stronger and more significant at mid- and long-term scales. A partial-wavelet coherence analysis underlines the intermediating role of economic policy uncertainty (EPU) in provoking the Bitcoin-S&P500 nexus. These results reflect the co-movement between US stock and Bitcoin markets beyond the second moment of return distribution and across time scales, suggesting the relevance and importance of considering fat tails and return asymmetry when jointly considering US equity-Bitcoin trading or investments and the policy formulation for the sake of US market stability.

## 1. Introduction

The Bitcoin market thrives on volatility. Notably, the frequent manifestation of Bitcoin’s boom and bust periods [1] suggests that variance cannot fully measure risk because it treats losses and gains equally and fails to differentiate downside risk probability from upside risk probability. In fact, Bitcoin returns are skewed, heavy-tailed, and deviate largely from the normal distribution [2], and the picture of the US stock market returns is not much different [3,4]. Under extreme market conditions, large shocks can provoke excess kurtosis, which in turn agitate the skewness away from the zero mark and intensify the variance. This phenomenon can spread from one market to another, emphasizing the relevance of considering higher-order moments to understand asymmetric or fat tail risk spillovers associated with extreme or downside (upside) risks [4,5]. This is relevant to the US stock and Bitcoin markets given evidence on the growing linkages between these two markets, especially under crisis periods such as the pandemic [6–8]. Accordingly, limiting the analysis of co-movement between Bitcoin and the US stock markets to the second moment (i.e. variance) would leave behind important connections reflected by the return asymmetry and extreme events.

The few existing studies focusing on the skewness and kurtosis of Bitcoin [9–11] provide no evidence on the co-movement between Bitcoin and the S&P500 based on higher-order moments. The debate on financial markets has been recently moved beyond the second moment (i.e., volatility) by considering higher-order moments, namely skewness and kurtosis [11,12]. Skewness captures the asymmetry of the returns distribution and is a proxy of tail or crash risk [13]. Kurtosis reflects the tail and peak features in the return distribution, where high kurtosis suggests large chances of facing extreme return values. Interestingly, the higher moments are very relevant to asset pricing [5,12] and portfolio allocation [14]. Related studies examine return and volatility dynamics and generally make inferences supporting the hedging and safe-haven role of Bitcoin for the US stocks [15–18], which arises from Bitcoin’s decentralization feature, detachment from the global financial system, and unique factors that drive its price dynamics [19]. During the pandemic, Kristoufek [20] challenges the safe-haven property of Bitcoin, and Conlon and McGee (2020) argue “S&P500 and Bitcoin move in lockstep, resulting in increased downside risk for an investor with an allocation to Bitcoin”. Kwon [7] shows that the tail behavior of Bitcoin can be related to that of the US stock market. Notably, Kumar et al. [8] point to stronger return and volatility linkages between Bitcoin and US stock markets in the post-pandemic period, suggesting that these two markets are no more detached, but can pose a contagion risk to the financial system.

Motivated by the above discussion and research gap, we enrich the academic finance literature by examining the co-movement between Bitcoin and S&P500 in higher-order moments, covering conditional volatility, skewness, and kurtosis. We do this within an analysis covering the time-frequency domain, which serves two reasons: Firstly, the time-domain analysis helps uncovering the time evolution of the co-movement, reflecting the effects of different crisis and boom periods on the co-movement between the two markets. Secondly, the frequency-domain analysis helps accounting for the heterogeneity of market participants in Bitcoin and the US stock markets, as indicated by the long-term investment horizons of institutional investors such as mutual funds and hedge funds and the short-term investment horizons of speculators and traders [18]. They highlight the importance of the time-frequency domain when considering Bitcoin and stock markets, and the heterogeneous investments horizons of participants in the markets of Bitcoin and US stocks.

Employing daily data from 19 August 2011 to 14 January 2022, we first model the time-varying conditional volatility, skewness, and kurtosis of Bitcoin and S&P500 returns using the GARCH with skewness and kurtosis (GARCHSK) model of León et al. [21], which accounts not only for stylized facts in the return series under study, such as volatility clustering, high kurtosis, heavy-tails, and slow-decaying autocorrelation function of squared-returns, but also allows for modelling the time-varying high-order moments. We then study co-movements via wavelet and partial-wavelet coherence methods, which allow for capturing the co-movements both in time and across scales.

The current study contributes to the academic literature in two aspects. Firstly, it extends the rising debate on the dynamics of market relationship between the US equity and Bitcoin markets beyond return and volatility [8,15,18,22–24] to the next level by reflecting potential co-movement in the third and fourth moments of return distribution. This is important, as both markets have been highly subject to turbulent and extreme events and co-jumping behaviors (e.g. Dutta and Bouri [25] indicate the presence of time-varying jumps in the cryptocurrency markets, whereas Xu et al. [26] show evidence of co-jumps between the cryptocurrency and the US technology sector.), which makes the analysis of second moment of returns suboptimal under the non-normality of the returns [2]. This opens the door for the consideration of the portfolio and risk inferences while accounting for fat tails and return asymmetry [14]. Secondly, our current study accounts for the heterogeneity of market participants in the US stock and Bitcoin markets by differentiating between (1) traders operating at short-term horizons ranging from few days to few weeks to long term investors such as fund managers and (2) institutional investors operating at long-term investment horizons exceeding three months [18,27]. Such a heterogeneity is captured by the wavelet-based analysis through which the co-movement is decomposed across various frequencies reflecting scales ranging from one day to more than a year (e.g. 256 days). This is important as the equity and Bitcoin markets comprise both types of market operators such as traders/speculators and investors/portfolio managers [20]. In that sense, we add to previous studies considering the relationship between the two markets in the time domain only [22,24,28], and those considering the frequency domain in the first and second moments only [18]. As such, an analysis of co-movements into the frequency-based relationship up to the fourth moment of returns would provide a more informative analysis to the various participants in the US equity and Bitcoin markets.

The main results show that the co-movement between Bitcoin and S&P500 is moment-dependent and varies in the time-frequency space. The co-movements in volatility intensify around the pandemic, especially at mid-term scales. The co-movements in skewness and kurtosis are stronger and more significant at mid- and long-term scales. A partial-wavelet coherence analysis underlines the intermediating role of the economic policy uncertainty EPU in provoking the co-movement between Bitcoin and US stock markets. From the portfolio perspective, Bitcoin has lost or at least considerably weakened its potential as a diversifier or a safe haven after 2018.

The rest of the paper is structured as follows. Section 2 reviews the related literature on the relationship between the US and Bitcoin markets. Section 3 describes the methods. Section 4 provides the data. Section 5 presents and discusses the results. Section 6 concludes the paper and provide some policy implications.

## 2. Literature review

Since the inception of Bitcoin in 2009 and its emergence as a new digital asset, a heated debate has emerged regarding the formation and dynamics of Bitcoin prices, suggesting the important role played by technological innovations (e.g. blockchain) and attractiveness, as measured by Google trends [19,29]. Later studies examine the relationship between Bitcoin and conventional assets, especially equities, in the largest economy, the US, indicating the detachment of the Bitcoin market from the global financial system and the diversification possibilities (see, among others, [15,22,28]), especially under stress periods. In this regard, the pioneering work of Bouri et al. [22] provide first empirical evidence on the hedging and safe-haven property of Bitcoin for specific asset and markets and a large strand emerges afterwards, using a large set of methods and data. Despite the richness of this strand of literature on the safe-haven and hedging properties of Bitcoin, which continues to grow in size and focus, it mainly considers return and volatility linkages between Bitcoin and equity markets [6,15,17,20,22,24]. However, the occurrence of several stress and crisis periods and departure of the returns of both markets from the normal distribution leave doubt on whether the above-mentioned findings are comprehensive, or they should be extended to the universe of higher-order moments. This also arises from evidence from the equity market literature highlighting the utility of considering higher-order moments for the construction of equity portfolios and hedging downside risk, especially under extreme events and crisis periods [14]. This is also the case of the highly volatile Bitcoin market, where extreme events and deviation from the normal return distribution are dominant stylized features.

On a related front, most of the above-mentioned studies overlook the frequency domain, despite the presence of a variety of participants in equity and Bitcoin markets, such as short-term and long-term investors covering traders/speculator and institutional investors, respectively [18]. Thus, it is relevant and necessary to account for such heterogeneity when studying co-moments between markets and assets, which helps in making decisions that are more appropriate across the time scales. In this paper, we study the time-scales co-movement between S&P500 and Bitcoin in higher-order moments, specifically up to the fourth conditional moment, an unexplored research topic.

## 3. Methods

### 3.1. Conditional higher-order moments of returns

GARCH-based modelling is an important tool to analyze financial data while accounting for volatility clustering. Standard GARCH models estimate the time-variation in the second moment of the return distribution, while assuming that the third and fourth moments are constant. In this section, we estimate and extract the time-varying conditional volatility, skewness, and kurtosis using the GARCHSK model proposed by León et al. [21] and later employed by Kräussl et al. [30]. This model allows for modelling the excess of volatility shocks when the proxy for actual volatility is not fully modelled by standard GARCH processes. As such, it captures asymmetry and excess kurtosis, which is very suitable to the context of the extremely highly volatile Bitcoin market and volatile stock market. Specifically, time-varying and conditional higher-order moments are obtained based on the GARCHSK process with the assumption that error terms follow a distribution of Gram-Charlier series expansion of the normality [21]. Accordingly, it relaxes the strict and somewhat assumption that third and fourth moments, which is realistic given the frequent occurrence of extreme events and shocks and the strong deviation of returns from the normal distribution. The GARCHSK model has been applied in numerous studies covering energy and stock markets [31,32]:

$$\begin{array}{l} \;r_{t}=r_{t-1}+\varepsilon_{t};\varepsilon_{t}∼(0,\sigma_{\varepsilon }^{2})\\ \varepsilon_{t}=h_{t}^{1/2}\eta_{t};\eta_{t}∼(0,1);\varepsilon_{t}|I_{t-1}∼(0,h_{t})\\ \;h_{t}=\alpha_{0}+\alpha_{1}\varepsilon_{t-1}^{2}+\alpha_{2}h_{t-1}\\ \;s_{t}=\beta_{0}+\beta_{1}\eta_{t-1}^{3}+\beta_{2}s_{t-1}\\ \;k_{t}=\delta_{0}+\delta_{1}\eta_{t-1}^{4}+\delta_{2}k_{t-1} \end{array}$$ (1)

where *r*_*t*_ is the log-returns of the index under study (i.e., Bitcoin or S&P500), and *h*_*t*_ is the conditional variance that follows a GARCH (1,1) structure. Notably, the results remain unchanged when we used the asymmetric GARCH (1,1) model. Furthermore, *s*_*t*_ and *k*_*t*_ denote the skewness and kurtosis corresponding to the conditional distribution of the standardized residuals $\eta_{t}\;(\eta_{t}{=\varepsilon }_{t}h_{t}^{-1/2})$. Using the Gram–Charlier series expansion, we estimate the model and truncate at the fourth moment. For more details, refer to [21,30].

### 3.2. Wavelet coherence

Having modelled and extracted the conditional volatility, skewness, and kurtosis of Bitcoin and S&P500 returns via the GARCHSK, we study the pairwise co-movement for each moment within a wavelet coherence approach allowing for capturing it in the time–frequency domain (Grinsted et al., 2004). The wavelet coherence method has been applied in empirical finance [27,33] and other research fields [34,35]. Importantly, the suitability and power of the wavelet coherence to the context of equity and Bitcoin markets cannot be overstated for several reasons. Firstly, it captures both positive and negative co-movements between variables across time and frequencies, and allows for making inferences regarding Granger causality flows. Secondly, it accounts for the heterogeneity of market participants such as traders and institutional investors. The former operate at short scales ranging from one day to a couple of weeks. The latter operate at longer investment horizons reaching several months and sometimes few years. Thirdly, through the partial wavelet coherence, one can capture “pure” correlation between the US equity and Bitcoin markets by excluding the effect of a control variable such as EPU.

#### 3.2.1. Wavelet coherence

Starting with the cross-wavelet transform between two time series, x(t) and y(t), it is given by:

$$W_{x,y}(u,s)={W_{x}(u,s)W_{y}}^{*}(u,s)$$ (2)

where, ’s’ is the scale index, ’u’ is the position index, and * denotes the complex conjugate.

The squared wavelet coherence *R*^2^(*u*, *s*) captures significant positive comovement through cross-wavelet power series at each scale. A value close to 0 (1) indicates a weak (strong) co-movement.

$$R^{2}(u,s)=\frac{|S[s^{-1}W_{x,y}(u,s)]{|}^{2}}{S[s^{-1}|W_{x}(u,s){|}^{2}]S[s^{-1}|W_{y}(u,s){|}^{2}]},\;with\;R^{2}(u,s)\in [0,1]$$ (3)

where, *S* denotes the smoothing parameter.

Since the wavelet coherency is squared, it does not indicate whether the relationship between the series is positive or negative. Interestingly, the wavelet coherence phase difference can capture both positive and negative comovements.

$$\rho_{x,y}(u,s)={tan}^{-1}\left(\frac{lm\{S(s^{-1}W^{x,y}(u,s))\}}{Re\{S(s^{-1}W^{x,y}(u,s))\}}\right),\;with\;\rho_{xy}\in [-\pi ,\pi ]$$ (4)

where *lm* is the imaginary smoothed part, and *Re* is the real part of the smoothed cross-wavelet transform.

We illustrate the results of wavelet coherence and phase difference in a map, in which the frequency is on the vertical axis (Short-term refers to the 1–4-day bands; medium-term refers to 4–8-day and 8–16-day bands; and long-term refers to 16–32-day bands. These patterns detected in the map can also reflect the causality relationship between the two series [36]. For example, the arrow points to the right when *x(t)* and *y(t)* are in-phase (or positively related). Conversely, the arrow points to the left when *x(t)* and *y(t)* are anti-phase (or negatively related). Furthermore, arrows can also reflect leads and lags relationships. For example, the arrow points left and up or right and down when *y(t)* leads *x(t)*. Conversely, the arrow points left and down or right and up when *x(t)* leads *y(t)*. On a different front, warm red (blue) colors represent regions with higher (lower) relationship. In this regard, black colored curves show the black contours of statistically significant coherence results at the 5% level. Finally, the u-shaped white solid line represents the region impacted by edge effects.

#### 3.2.2. Partial wavelet coherence

Partial correlation [37] allows for capturing more “pure” correlation between between *x(t)* and *y(t)* by excluding the effect of a control variable z(t).

$${R_{p}}^{2}(x,y|z)=\frac{{\left|R(x,y)-R(x,z)⦁{R(x,y)}^{*}\right|}^{2}}{{\left[1-R(x,z)\right]}^{2}{\left[1-R(y,z)\right]}^{2}}\;with\;{R_{p}}^{2}(x,y,z)\in [1,0]$$ (5)

where *R*(*x*, y), *R*(*x*, z), and *R*(*x*, y) are transfer wavelet coherence between *x* and *y*, *x* and *z*, and *x* and *y*, respectively. The asterisk indicates the complex conjugate. The confidence intervals of the wavelet and partial wavelet coherences are estimated based on Monte Carlo methods.

*R*_*p*_^2^(*x*, *y*, *z*) has a similar interpretation as *R*^2^(*u*, *s*). Bitcoin and S&P500 can be influenced by EPU. Therefore, comparing the levels of cross-wavelet transform and partial wavelet transform captures the intermediating role of EPU in provoking the co-movement between the two markets. If the level of *R*_*p*_^2^(*x*, *y*, *z*) is substantially lower than the corresponding *R*^2^(*u*, *s*), then the co-movement between the *x(t)* and *y(t)* is facilitated by their respective co-movements with *z(t)*.

## 4. Data

**Fig 1:**
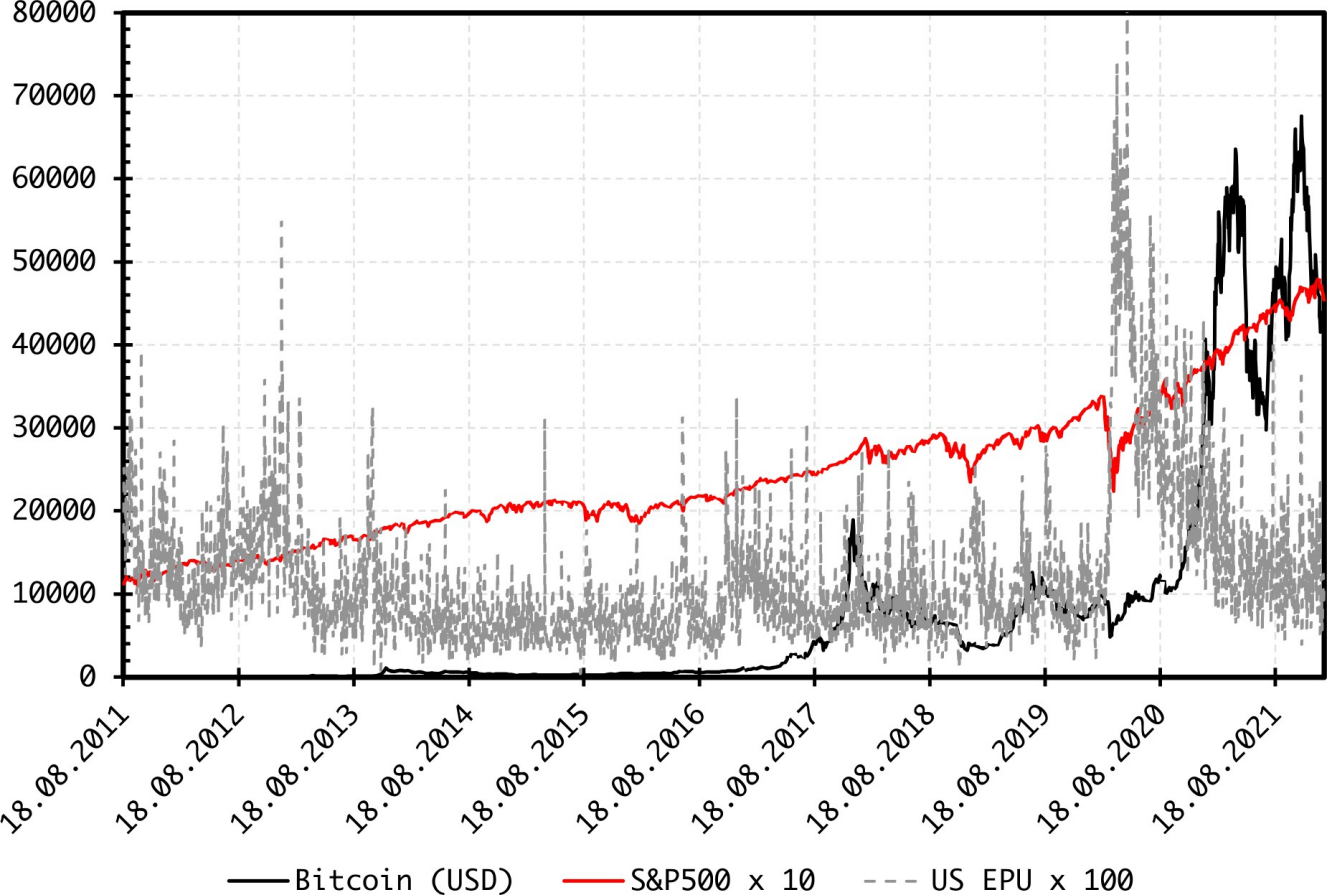
Plots of levels.

Collected from DataStream, daily price data on Bitcoin and S&P500 index are from 19 August 2011 to 14 January 2022, yielding 2,716 daily observations. The beginning of the sample period is dictated by the availability of Bitcoin price data from Bitstamp, one of the well-established and largest Bitcoin exchange. We use Bitcoin price against the USD. Fig 1 shows that the evolution of Bitcoin and S&P500 levels is somewhat synchronized. Notably, large fluctuations occur around the COVID-19 outbreak, after which the two series spiked, yet exhibited some disparity in the levels and intensity of corrections. This might indicate potential co-movements in the various moments at the time-frequency domain, which can be captured via wavelet coherence.

We also use the daily index of the US EPU [38,39] downloaded from https://www.policyuncertainty.com/ and plotted in Fig 1 as well. The EPU can affect the market dynamics of both Bitcoin and S&P500 [39], and thereby we account for it when analyzing the co-movements between each moments of Bitcoin and S&P500. The level of the EPU increased sharply in February-March 2020, around the COVID-19 outbreak.

**Fig 2:**
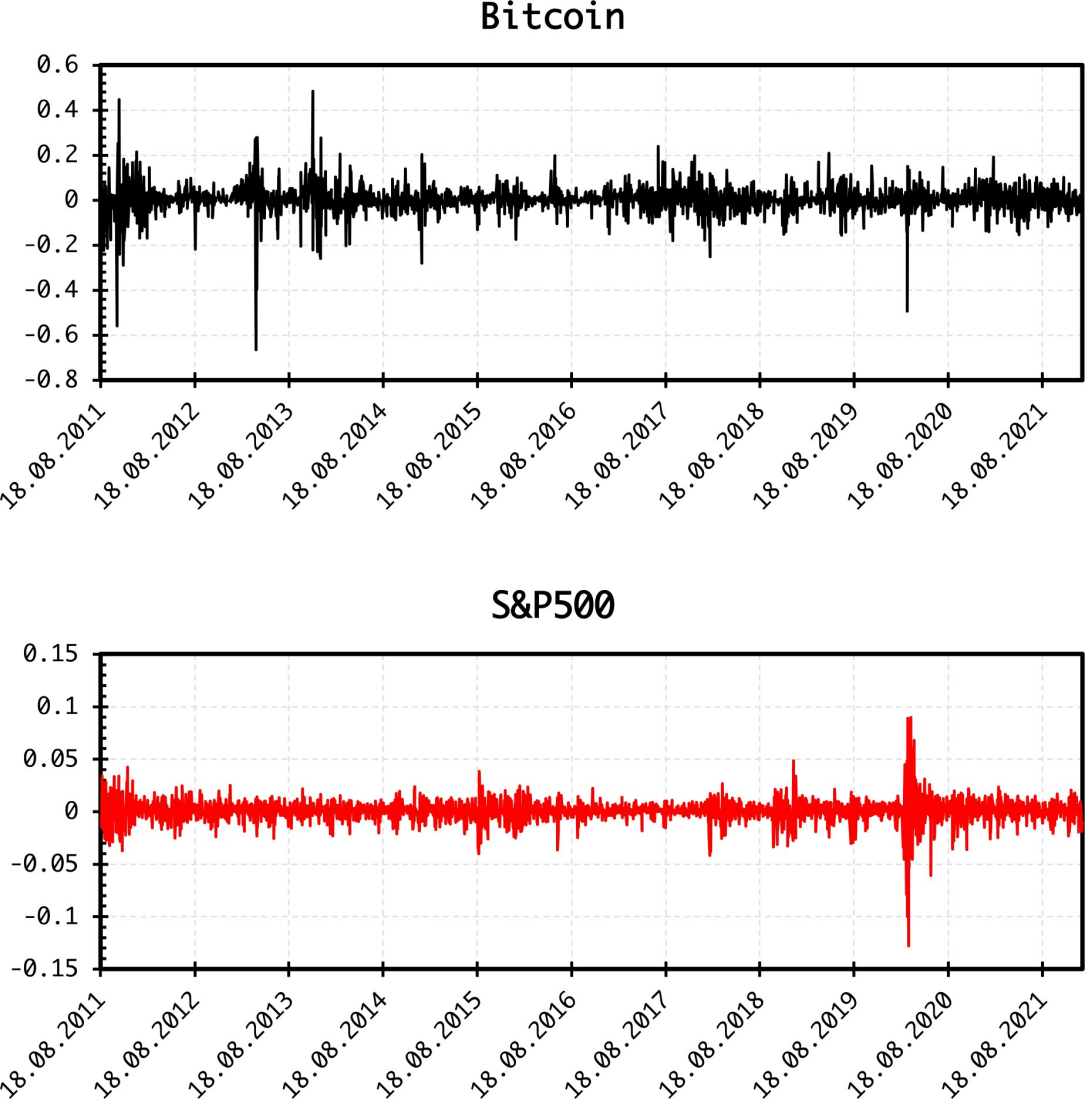
Plots of daily returns.

The daily log returns are plotted in Fig 2, showing large fluctuations for Bitcoin, whereas both return series exhibit large variations around the COVID-19 outbreak. Summary statistics of log returns (Table 1) indicate abnormal distribution. Bitcoin returns are more volatile than S&P500 returns. Skewness values are negative and excess kurtosis dominates. Both return series are stationary at the 1% level of significance. The same is true for the EPU levels.

**Table 1:** Summary statistics.

	Mean	Min.	Max.	Standard Deviation	Skewness	Kurtosis	Jarque-Bera	Phillips-Perron	Augmented Dickey-Fuller
Bitcoin	0.003	0.485	-0.664	0.057	-1.067	23.134	46442.091***	-54.825***	-54.890***
S&P500	0.001	0.090	-0.128	0.010	-0.882	22.571	43744.549***	-60-471***	-17.153***
EPU	118.741	3.320	807.660	90.156	2.511	11.758	11.553***	-28.185***	-4.614***

Note: This table shows the summary statistics and stationarity results of the returns of Bitcoin and S&P 500 and the levels of the EPU index. The sample period is 19 August 2011 to 14 January 2022.

*** denotes statistical significance at the 1% level.

## 5. Results and discussion

### 5.1. Extraction of the conditional volatility, skewness, and kurtosis

**Fig 3:**
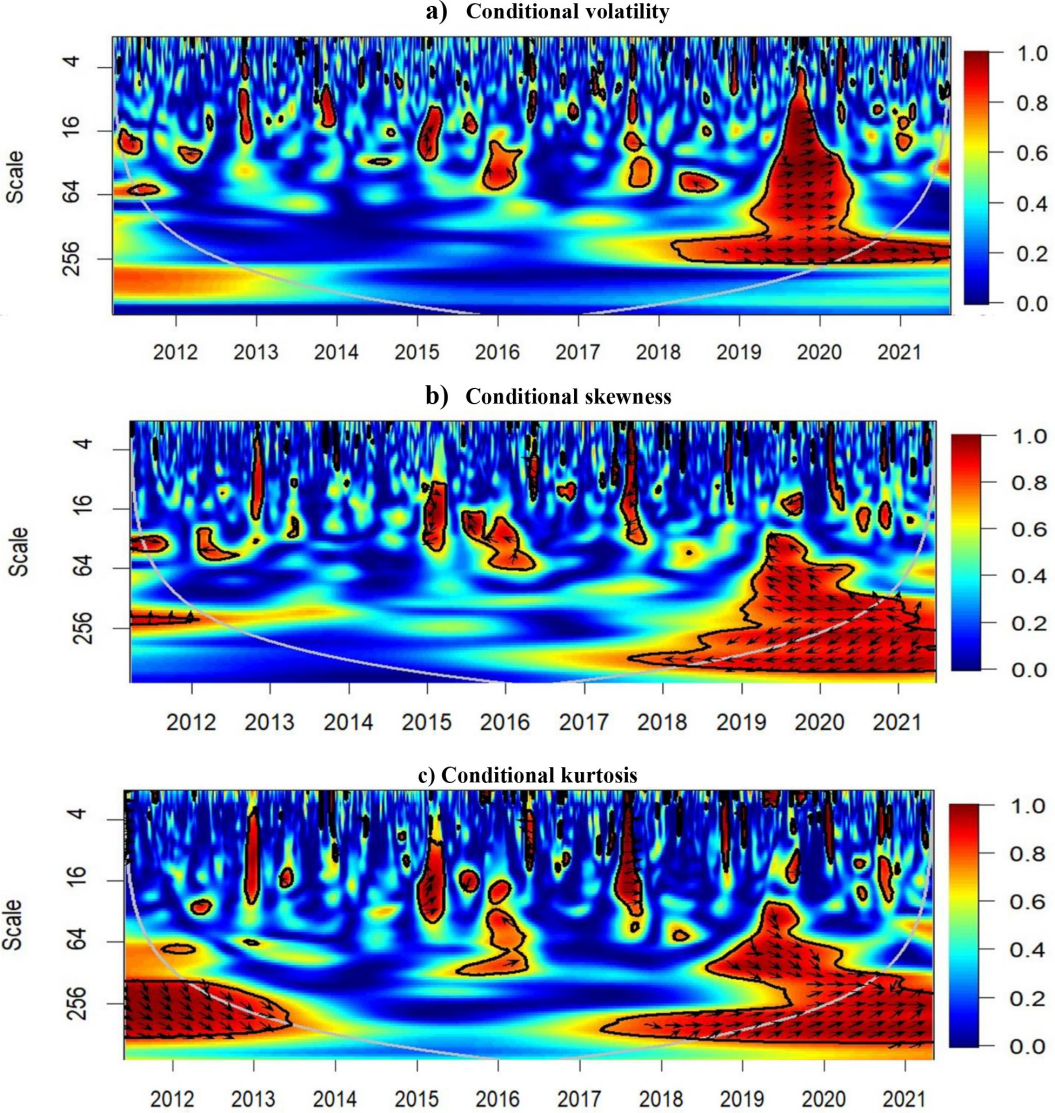
Results of wavelet coherence–Bitcoin and S&P500. a) Conditional volatility b) Conditional skewness c) Conditional kurtosis.

Our main focus is to use the conditional higher-order moments within a wavelet coherence analysis. Therefore, we do not extensively analyze the output of the GARCHSK model, bearing in mind that the coefficients of the GARCHSK are generally significant at the conventional levels. More details on the estimated coefficients of the GARCHSK models are available from the authors upon request. Table 2 presents a summary of the statistics of the conditional moments for both Bitcoin and S&P500. As expected, Bitcoin is more volatile than S&P500 and even the lowest reported conditional volatility for Bitcoin is much higher than the average conditional volatility for the stock index. The S&P500 index is on average more negatively skewed, which is rather unexpected. However, the conditional skewness of Bitcoin experiences much larger swings than the conditional skewness of the stock index. The maximum conditional volatility for Bitcoin is even as much as two orders of magnitude higher than for S&P500, which represents large upward swings in the cryptocurrency while also the largest downward swings are much more pronounced for Bitcoin. Still, the average level of conditional skewness is on similar levels, considering the large uncertainty in the cryptocurrency. Similar story is told for the conditional kurtosis with comparable average levels but much more unstable and swingy ones for the cryptocurrency. Appendix Fig A.1 in S1 Appendix shows the time-evolution in the extracted conditional movements of Bitcoin and S&P500 returns. We notice a large spike in the volatility of both indices around the COVID-19 outbreak (See Fig 3(A)), although Bitcoin experienced much higher volatility around late April 2013. During that period, Bitcoin price dropped from over $260 to around $50 and then rose to around $100, driven by concerns over the processing delays in the payment by BitInstant and Mt. Gox. From the visual perspective, it seems that prior to 2017, Bitcoin volatility had been driven mostly by unique exogenous shocks, or more precisely shocks that had not been shocks to the general stock market represented by the S&P500 index. However, the spikes, albeit of different magnitudes, seem to move together following 2017. Fig 3(B) shows a spike in its positive skewness around the COVID-19 outbreak of mid-March 2013, resulted from the big rebound of Bitcoin price following its correction during the peak of the pandemic. During the same period, S&P500 mostly experienced large fluctuations in both directions, which resulted in more negative skewness values, indicating a high level in the crash risk. The reaction to the new wave of quantitative easing following the confirmation and realization that COVID-19 is a global pandemic has reflected differently on the two markets. Fig 3(C) exhibits the excess values of kurtosis for both series around the March 2020 period.

**Table 2:** Summary statistics of the three conditional moments.

	Mean	Median	Maximum	Minimum	Std. Dev.	Skewness	Kurtosis	Jarque-Bera
Panel A: Conditional volatility								
Bitcoin	31.780	20.656	587.771	8.065	43.971	6.699	64.638	450263
S&P500	0.732	0.518	13.106	0.352	0.943	8.553	88.349	857481
Panel B: Conditional skewness								
Bitcoin	-0.072	-0.124	87.889	-19.648	2.303	29.977	1073.931	130000000
S&P500	-0.213	-0.187	0.662	-2.924	0.181	-7.043	75.929	624356
Panel C: Conditional kurtosis								
Bitcoin	4.275	3.891	122.609	3.862	3.760	21.567	552.300	34356429
S&P500	3.647	3.425	22.600	3.376	0.963	9.345	120.827	1610635

Notes: This table shows the summary statistics of the conditional volatility, skewness, and kurtosis, modelled via the GARCHSK model. The sample period is 19 August 2011 to 14 January 2022.

### 5.2. Results of wavelet coherence

The results of the wavelet coherence between each moment of Bitcoin and S&P500 returns are presented in Fig 3. Starting with Fig 3(A), the conditional volatility of Bitcoin and that of S&P500 are rather detached before 2018 with only several islands of significant co-movement. Such tiny islands can be easily artifacts of the procedure as the confidence intervals are generated by the Monte Carlo simulations. Nevertheless, the connection of conditional volatilities after 2018 is evident. As a reminder–the right-pointing arrow signifies positive co-movement and the upward pointing one directs towards the first series leading the other. From 2018, the uncertainty in both the stock market and the cryptomarkets started building up and culminated at the break of 2019 and 2020 going through practically all analyzed scales. At the break and in Q1 2020, Bitcoin became a weak leader in the relationship, which means it reacted to the situation earlier than the S& 500. The uncertainty thus started building up faster in the cryptocurrency which does not connect well to its desired property as a safe haven against uncertainty in the stock markets. From Q2 2020 onwards, the conditional volatilities have been detaching from one another, even though they kept their connection on higher scales, i.e., on the longer investment horizons around one year. Importantly, the connection is positive, which again goes against the safe haven narrative. Interestingly, the connection drops rather quickly after Q1 2020, signifying that the monetary injections in reaction to the COVID-19 pandemic had different effects on the two markets or at least had effects of different magnitudes. Even though both markets reacted positively to the inflow of new money, S&P500 doubled from around 2,500 points in Q1 2020 to 4,800 in Q4 2021 whereas Bitcoin went from a low around 5,000 USD to over 60,000 USD, i.e., a twelve-fold rally, in the same two time points, but with several periods of pronounced high(er) volatility. The timing is important as it seems that a structural break occurred in 2018 onwards, i.e. even before the COVID-19 pandemic and the later financial stimuli, and did not disappear after the monetary injections started fizzling their effect. As volatility and its co-movement is essential for potential portfolio utility and overall portfolio risk optimization, Bitcoin has lost much of its appeal from 2018 onwards, at least from the long-term perspective. For short holding periods in a portfolio, Bitcoin might still serve as a diversifier, even though its position was markedly weakened during the early periods of the COVID-19 pandemic and the induced financial turmoil. Notice that at the higher scales, the arrow direction straightens and becomes practically horizontal which means that none of the assets is a leader, their volatility simply moves together, likely reacting to the same news or global financial shocks, not to one another. This only further supports the weakening utility of Bitcoin as a diversifier in a standard portfolio as reacting to the same news or shocks is the exact opposite of what a good diversifier should do.

Regarding the conditional skewness, Fig 3(B) shows that the small islands of significance for conditional volatility are not statistical artifacts but are more likely connected to specific shocks during the time periods as these overlap for the conditional skewness and volatility while being more pronounced for the former. Interestingly, when the co-movement of the conditional skewness is statistically significant, the arrows are mostly oriented to the left, suggesting a negative correlation between the two series. For the islands before 2018, it is rather difficult to identify who the leader is, not only because of the short period of significance but also due to the arrow almost directly pointing downwards where it can easily flip from both negative to positive connection and the lead-lag relationship. The picture is clearer for the period starting in 2019. Within the cone of influence, arrows pointing to the left, i.e., negative correlation, mostly in the north-west direction, signify the S&P500 is the leader. Going back to Fig A.1(B) in S1 Appendix, we see that this period is mostly covered by spike to the negative areas of conditional skewness for the stock index and a large spike of positive skewness for the cryptocurrency, which seems to be dominating the relationship in the period. However, even without the spike for Bitcoin, we see that S&P500 experienced quite frequent downward jumps of conditional skewness whereas the one of Bitcoin remained rather stable, at least within the scale Bitcoin went through. As Bitcoin’s skewness did not react to the negative jumps in S&P500, the negative correlation emerged. Therefore, although it might be rather counterintuitive, or unexpected to say the least, the stock index experienced a more jumpy dynamics in the pandemic period than the cryptocurrency within their respective scales. Fig A.1 in S1 Appendix confirms that S&P500 experienced a higher number of downward jumps of its conditional skewness compared to Bitcoin, even though the scale of each series is rather different with Bitcoin reaching much higher values but mostly in the positive region of the conditional skewness. Interestingly, the overall connection with respect to the conditional skewness does not go through the whole spectrum of scales as in the conditional volatility but is rather more representative for higher scales. Either way, year 2018 is again identified as a starting point of the structural change in the relationship between the two financial assets. However, contrary to the results of co-movements of conditional volatilities, the detachment of the realized skewness series suggests that Bitcoin could have its role as at least a weak safe haven as it does not move together with the stock index much in the extreme events. However, this needs to be confirmed by similar results for conditional kurtosis.

Fig 3(C) shows the conditional kurtosis connection and presents quite similar picture to the wavelet coherence dynamics and spread across scales as for the conditional skewness. However, the asymmetry of skewness, or rather the more frequent skewness jumps in S&P500 are hidden within the kurtosis symmetric nature. The connection between the stock index and the cryptocurrency is thus positive and the former mostly leads the relationship from 2018 onwards, and from 2019 onwards over a larger spread of scales. The extreme movements thus generally come together but mostly the stock index leads the way. This is not in hand with the results for the conditional volatility, which induces an interesting situation when the extreme movements are mostly driven by the stock index dynamics but the overall level of uncertainty is more swiftly transmitted into the cryptomarket. The above results indicate significant co-movements in both higher-order moments [11] and time-frequency [33], reflecting the interlinkages of return asymmetry and extreme events which matter for participants in both Bitcoin and US stock markets. But how to interpret the results for conditional skewness and conditional kurtosis that seemingly go against one another. Skewness describes the general asymmetry, it focuses on the whole side of the distribution, not just the most extreme movements. Otherwise, there would be no need for kurtosis. Bitcoin can thus serve as rather weak safe heaven when the swings in the market are not extreme. Bitcoin would not react, or not strongly, when the stock market moves mildly from its mean behavior. However, in the extreme cases when the swings go into the tails of the distribution, Bitcoin moves together with the stock market. This connection again emerges after 2018 and mostly at higher scales, meaning that at the high frequencies, one might still utilize Bitcoin portfolio attributes. However, in the medium term up, Bitcoin mostly converges to the traditional financial market, here represented by the S&P500 index.

### 5.3. Results of partial wavelet coherence

**Fig 4:**
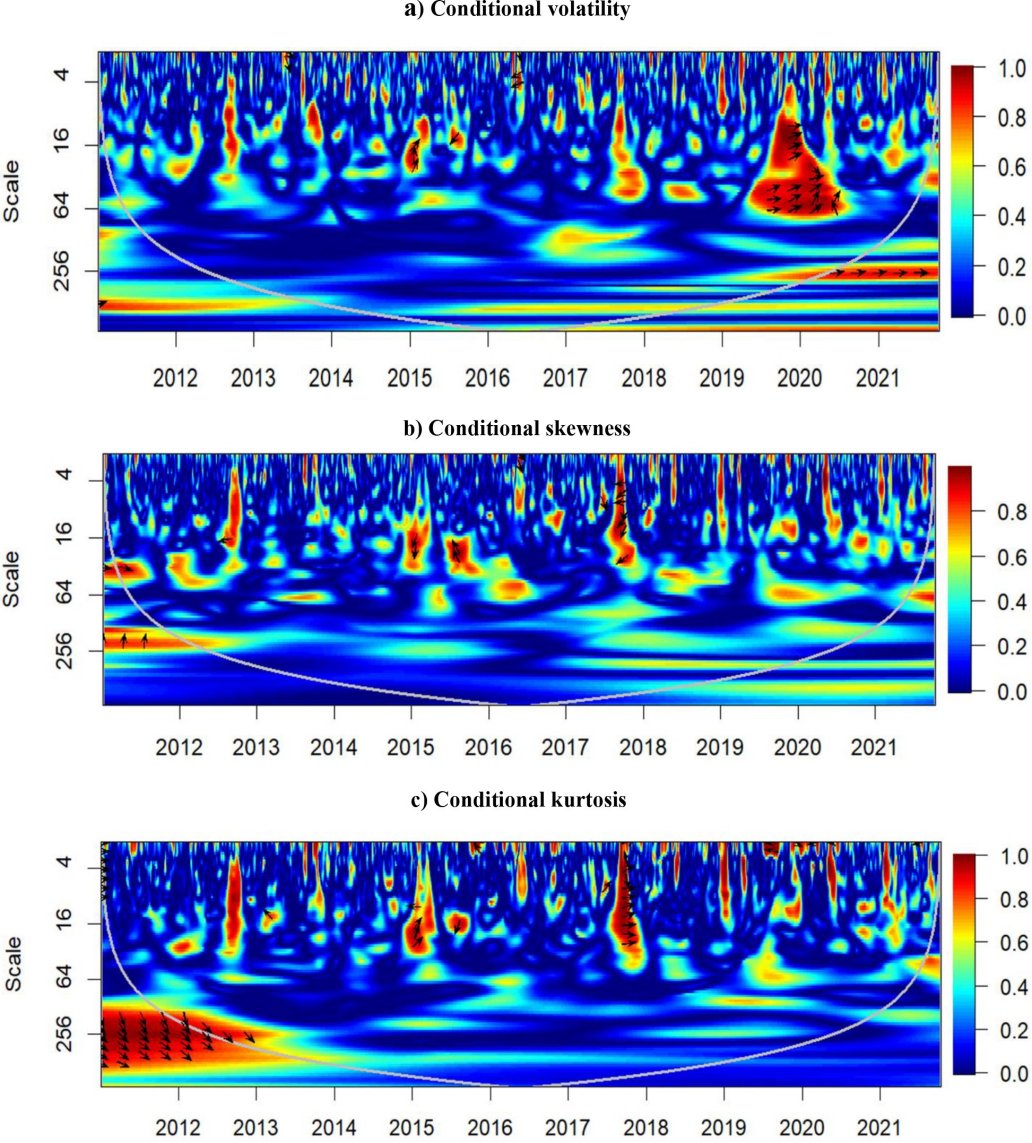
Results of partial wavelet coherence- Bitcoin and S&P500⎹ EPU. a) Conditional volatility b) Conditional skewness c) Conditional kurtosis.

As a robustness analysis and to see how much of the reported dynamics is due to overall market uncertainty, we account for the role of the EPU via the application of the partial wavelet coherence and present the results in Fig 4. Overall, they are different from those presented in Fig 4, for all three moments. Fig 4(A) shows that even though a large portion of the correlation between the conditional volatility is gone, there remains clear positive co-movement with a weak leadership of Bitcoin. However, most of the connection between the higher moments is gone after controlling for EPU (Fig 4(B) and 4(C)), which partially concord with [38,39]. Practically, the only connection that remains is during the cryptomarket reversal at the breaking of 2017 and 2018, which is also linked to the stock market slowing down (the S&P500 added around 40% in 2017 but became mostly stagnant over 2018 with a correction of around 20% by the end of 2018). The results are almost identical when VIX is considered instead of EPU. Although, the VIX seems to take away more of the connections from the original wavelet coherence, signaling that the financial market uncertainty is likely more important for the connection between Bitcoin and S&P500 than the general economic uncertainty. This confirms the explanation and interpretation presented in the previous section that Bitcoin and S&P500 react similarly to the financial news from 2018 onwards, weakening the utility of Bitcoin as either diversifier or safe haven for the traditional portfolios.

## 6. Conclusions

Not only do the conditional second moment of S&P500 and Bitcoin evolves quite dramatically over time but so do the relationships between their respective higher-order moments in the time-frequency domain. Before 2018, the two markets were mostly detached with respect to their common dynamics in volatility, skewness, and kurtosis. In 2018, and mostly in 2019 onwards, they have become more interconnected. The interplay between the different moments shows an interesting picture of Bitcoin quickly absorbing the coming uncertainty and reacting to the financial news and shocks faster than the stock market. However, when it comes to extreme movements (rather than gradual building up of uncertainty), it is the stock market that reacts first and only then comes Bitcoin.

From a more general perspective, we have further validated some previous results on Bitcoin safe haven properties being mostly unfounded [6,28,40] as the basic dynamic properties of the stock markets and the cryptocurrency generally co-move during the turbulent period, mostly represented by the first quarter of 2020 before the unprecedented monetary injections came for the rescue. By controlling the common dynamics with the inclusion of the economic policy uncertainty index in our robustness check, we further confirm the claims that both markets react similarly to the global financial news and market shocks, both in the mild and extreme movements, making Bitcoin a weak safe haven to the traditional financial market at best, but mostly not even.

Further evidence shall come after the current military conflict between Russia and Ukraine which will likely not be directly followed by the monetary expansion as the inflation has already escalated the global economic tensions. We might thus come to another chance for Bitcoin to show whether the claims or wishful thinking of its safe-haven status had simply not been given enough time to propagate during the pandemic as the monetary stimuli came too quickly and overshadowed possible action. Or the link between the stock and cryptocurrency markets has been already established and Bitcoin has come much closer to becoming a standard financial asset, albeit very risky. We shall see in the coming months and years.

## Supporting information

S1 Appendix (DOCX)

S1 Data (CSV)
