# Supplementary material for: Bitcoin and S&P500: Co-movements of high-order moments in the time-frequency domain
Source: PLoS One. 2022 Nov 22;17(11):e0277924. doi: 10.1371/journal.pone.0277924 (PMC9681096; doi:10.1371/journal.pone.0277924)
Supplement: S1 Appendix — (DOCX) [file pone.0277924.s001.docx]

APPENDIX

**Figure A.1. Plots of the conditional volatility, skewness and kurtosis**

1. **Conditional volatility**

1. **Conditional skewness**

1. **Conditional kurtosis**

**List of abbreviations**

EPU Economic policy uncertainty (index)

GARCH Generalized autoregressive conditional heteroskedasticity model

GARCHSK Generalized autoregressive conditional heteroskedasticity model with skewness and kurtosis

S&P500 Standard & Poor’s 500 stock market index (ticker GSPC)

VIX CBOE (Chicago Board Options Exchange) Volatility Index (ticker VIX)
